# In Vitro Comparative Study of Calcium Hydroxyapatite (Stiim): Conventional Saline Dilution Versus Poly-Micronutrient Dilution

**DOI:** 10.7759/cureus.80344

**Published:** 2025-03-10

**Authors:** Valeria Dal Col, Cassiano Marchi, Fabio Ribas, Bibiana Franzen Matte, Helouise Medeiros, Beatriz Domenici de Oliveira, Renata Viana

**Affiliations:** 1 Dentistry, Protocoll Clinique, Vitória, BRA; 2 Dentistry, CM Harmonização Orofacial, São Paulo, BRA; 3 Research, Núcleo Vitro, Porto Alegre, BRA; 4 Biomedical Sciences, Independent Research, São Paulo, BRA; 5 Research, Private Practice, São Paulo, BRA

**Keywords:** calcium hydroxyapatite, collagen, elastin, fibroblasts, in vitro study, longevity gene expression, polymicronutrients

## Abstract

Calcium hydroxylapatite (CaHA) is widely used in aesthetics for its dual role as a dermal filler and biostimulator, promoting collagen and elastin synthesis. This study evaluates the comparative potential of CaHA in conventional saline dilution versus CaHA mixed with a poly-micronutrient solution (CaHA/PMN) to enhance its biostimulatory effects. In an in vitro model, primary human fibroblasts were treated with both formulations, and cell viability and gene expression of type I collagen, elastin, FOXO3, SIRT-1, and SIRT-3 were assessed. The results demonstrated that both CaHA and CaHA/PMN treatments significantly improved cellular responses compared to the control. CaHA/PMN outperformed conventional CaHA, with greater increases in cell viability, collagen synthesis, elastin synthesis, and the expression of SIRT-1, SIRT-3, and FOXO3. These findings suggest that poly-micronutrient-enriched solutions can enhance CaHA’s regenerative potential, providing a promising approach for skin rejuvenation and elasticity improvement.

## Introduction

The connective tissue of the skin is composed mostly of collagen and elastin. Collagen makes up 70-80% of the dry weight of the skin and gives the dermis its mechanical and structural integrity. Elastin is a minor component of the dermis, corresponding to 2-4% of the extracellular matrix, but it has an important function in providing the elasticity of the skin [1,2]. During aging, the synthesis and function of collagen and elastin gradually decline and clinically reflect the signs of aging.

Naturally aged and sun-induced photoaged skin share common molecular features, including accumulation of damaged dermal connective tissue collagen [3]. The fragmentation of collagen fibrils is the hallmark of skin dermal aging [3,4]. This alteration in collagen is a major contributing factor to clinical changes, such as fragile and wrinkled skin, which are prominent features of skin aging [5].

Calcium hydroxyapatite (CaHA) microspheres have been very well known in aesthetic medicine not only for dermal filling purposes but also as a biostimulator agent since it induces cell synthesis, increasing collagen and elastin proliferation [6-8]. More recently, CaHA is cited as one of the products used in regenerative medicine, aiming to restore the structure and functions of damaged or diseased tissues [7,9].

Recent studies have explored the addition of poly-micronutrient solutions relying upon establishing an adequate skin micronutrient profile to enhance the protein-synthesizing effect of CaHA [10].

This study aims to compare the biostimulatory potential of CaHA Stiim diluted in conventional saline versus a poly-micronutrient solution. We hypothesize that the addition of a poly-micronutrient solution enhances the regenerative effects of CaHA by increasing the stimulation of type I collagen and elastin production in human dermal fibroblasts, as well as upregulating key cellular longevity regulators, including FOXO3, SIRT-1, and SIRT-3. By identifying potential differences in cellular responses, this study seeks to determine whether poly-micronutrient enrichment provides a more favorable microenvironment for extracellular matrix remodeling and cellular longevity.

## Materials and methods

The study was designed as an in vitro experiment to evaluate and compare the potential of cellular responses of CaHA bio-stimulator using in vitro fibroblast cultures. It assessed the ability of the samples to stimulate cell proliferation and increase the expression of type I collagen, elastin, FOXO3, SIRT-1, and SIRT-3.

**Figure 1:**
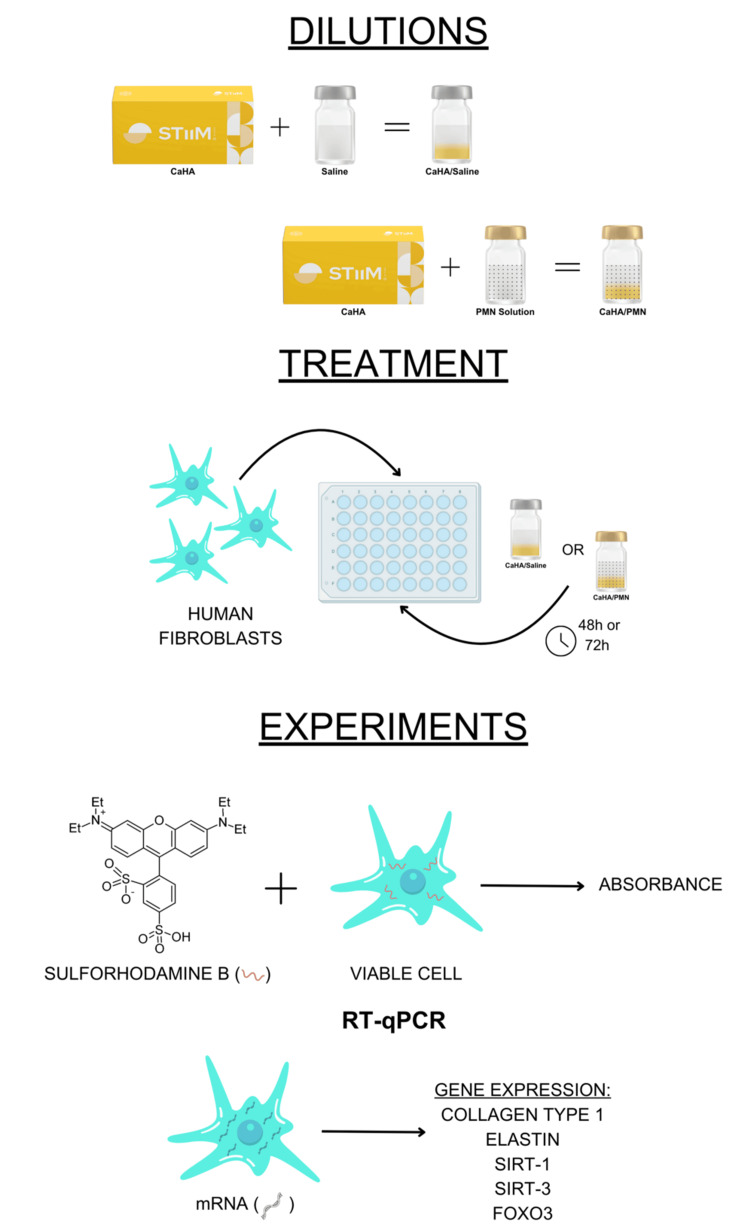
Overview of experimental design and methods Schematic representation created using Canva

The in vitro study evaluated and compared CaHA bio-stimulator in conventional saline dilution 1:1 versus CaHA diluted with a poly-micronutrient solution. The sulforhodamine B (SRB) assay was assessed at 48 hours. Primary human fibroblasts, isolated from human dermis tissue, were cultured and treated with each product, and gene expressions were measured using quantitative reverse transcription polymerase chain reaction (RT-qPCR) after 72 hours. Fibroblasts were obtained from a single donor to ensure consistency in experimental conditions. All experiments were conducted in triplicate to enhance reproducibility. A graphical summary of the study methodology is presented in Figure 1.

Cell culture

Primary cultures of human dermal fibroblast cells were isolated from human dermis tissue, approved under CAAE#59124916.6.0000.5327, and maintained in Dulbecco’s Modified Eagle’s Medium (DMEM, Gibco, USA) supplemented with Fetal Bovine Serum (FBS, Gibco, USA) to support optimal cell growth. The cultures were kept in a humidified incubator at 37 °C with 5% CO_2_, providing a controlled environment to ensure cellular health and proliferation. Fibroblasts were obtained from a single donor to ensure consistency in the experimental conditions. All experiments were conducted in triplicate to enhance reproducibility. All handling procedures, including media changes and cell passaging, were performed under sterile conditions in a laminar flow hood to minimize the risk of contamination.

Products

Calcium Hydroxyapatite (Stiim)

STIIM is a biodegradable filler consisting of 30% calcium hydroxyapatite (CaHA) microspheres and 70% carboxymethylcellulose gel (Ilikia - CGBio, South Korea), presented in a pre-filled syringe containing 1.5 mL. The CaHA microspheres show a lattice pore structure, and it is designed for slow degradation and sustained biostimulation, enhancing fibroblast activity, stimulating collagen synthesis, and contributing to extracellular matrix regeneration [11]. The carboxymethylcellulose gel acts as a carrier, ensuring uniform distribution during application. CaHA is widely utilized in aesthetic and regenerative treatments to improve skin structure and elasticity through biostimulation, particularly when applied to the superficial subcutaneous layer [9].

Poly-Micronutrient Solution

The poly-micronutrient (PMN) solution used in this study was designed to enhance fibroblast activity and optimize extracellular matrix (ECM) remodeling. Its formulation includes bioactive peptides (Matrixyl, Syn-Coll, Syn-Hycan, Syn-TC, Syn-Tacks, Syn-UP, Syn-AKE), B-complex vitamins (thiamine, nicotinamide, pantothenic acid, pyridoxine, biotin, methylfolate, methylcobalamin), amino acids (glycine, lysine, proline), growth factors (IGF, EGF, FGF, TGF), and coenzyme (NADH).

The PMN solution works by providing essential biosynthetic substrates for fibroblasts, optimizing cellular energy production, and enhancing mechanical interactions with the ECM [12-16]. Many of these components have been shown to stimulate collagen synthesis, improve fibroblast function, and support skin homeostasis [17-19]. Bioactive peptides, such as Matrixyl and Syn-Coll, activate collagen synthesis via TGF-β signaling, while growth factors like IGF and TGF-β promote fibroblast proliferation and ECM remodeling [20-22]. Additionally, micronutrients such as zinc, magnesium, and inositol contribute to cellular metabolism and tissue repair [17,23].

Sample preparation and treatments

Primary human dermal fibroblasts at passage number 12 were seeded into 96-well culture plates at a concentration of 1 × 10³ cells per well, ensuring consistent distribution for subsequent analyses.

The following treatments were prepared with CaHA using two different dilutions. In the first treatment (CaHA), 10 mg of Stiim was dissolved in an equal volume of saline solution. For the second treatment (CaHA/PMN), 10 mg of Stiim was dissolved in an equal volume of a poly-micronutrient solution.

A control group was also prepared in a culture medium in a reduced-supplement culture medium. All solutions were freshly prepared immediately before the experiments to ensure consistency and stability.

After 24 hours of cell plating to allow cell adhesion, cells were treated with the cited solutions of CaHA diluted to a concentration of 0.5% with DMEM supplemented with fetal bovine serum (FBS), resulting in the final mixture with a total volume of 100 µL per well. Cells in the control group were maintained under identical conditions but received only DMEM supplemented with FBS. Cultures were incubated at 37 °C and 5% CO₂ for up to 48 hours. Cell viability, proliferation assays, and RNA extraction were performed at this endpoint. All tests were conducted in triplicate to ensure reproducibility, and no additional substances were added to the experimental setup.

Fibroblast viability

Cellular viability was assessed after 48 hours using the SRB assay. After medium removal, cells were fixed with 10% trichloroacetic acid (TCA) at 4 °C for 1 hour. The fixed cells were then washed with distilled water to remove residual TCA.

Subsequently, the cells were stained with 0.4% SRB in 1% acetic acid for 30 minutes at room temperature to allow binding to cellular proteins. Excess dye was removed by washing the wells with 1% acetic acid, and the plates were air-dried. The bound dye was solubilized with 10 mM Tris base solution (pH 10.5) under gentle agitation for 10 minutes.

Absorbance measurements were performed at a wavelength of 560 nm using a spectrophotometer. The absorbance values were directly proportional to the protein content, providing a quantitative measure of cellular viability.

Gene expression analysis

Gene expression of collagen type I, elastin, FOXO3, SIRT-1, and SIRT-3 was evaluated using reverse transcription polymerase chain reaction (RT-qPCR). After 72 hours of treatment, cell extracts from both the treated and control groups were collected for analysis.

mRNA extraction: Messenger RNA was extracted using Trizol reagent (Gibco, Waltham, Massachusetts, US). The quantity and purity of the extracted mRNA were assessed, with purity values ranging between 1.8 and 2.0 to ensure the quality of the samples for downstream applications.

cDNA synthesis: Complementary DNA (cDNA) was synthesized from 2000 ng of RNA in a reverse transcription reaction. The high-capacity cDNA reverse transcription kit (Thermo Fisher Scientific, Waltham, Massachusetts, US) was used, following the manufacturer’s instructions. Each reaction consisted of 10 μL of the cDNA synthesis mix, prepared with the necessary reagents for 2000 ng of RNA, and the remaining volume was adjusted with diethylpyrocarbonate (DEPC)-treated water.

Real-time polymerase chain reaction (RT-qPCR): RT-qPCR was conducted using the SYBR green reagents (Thermo Fisher Scientific), using specific primers for target genes and β-actin served as an endogenous control to normalize the results, ensuring accuracy and reliability of the expression data.

Specific primers for collagen type I, elastin, FOXO3, SIRT-1, and SIRT-3 were used. The primer sequences for each target gene were as follows: forward primer AGGGCCAAGACATC and reverse primer AGATCACGTCATCGCACAACA for collagen type I; forward primer GTATATACCCAGGTGGAGTG_ and reverse primer CGAACTTTGCTGCTGCTTTAG for elastin; forward primer AGAACTCCATCCGGCACA and reverse primer TACAAGTCAGCCGTGGCA for FOXO3; forward primer GCCTCACATGCAAGCTCTAGTGAC and reverse primer TTCGAGGATCTGTGCCAATCATAA for SIRT-1; forward primer CTTGTGCAGCGGGAAACT and reverse primer TCCTATGTTACCATTTATTGTGTGG for SIRT-3; and forward primer CCAGAGGCGTAGAGGGATAG and reverse primer CCAACCGCGAGAAGATGA for β-actin.

The reaction conditions included an initial denaturation step at 95 °C for 10 minutes, followed by 40 cycles of denaturation at 95 °C for 15 seconds, annealing at 60 °C for 30 seconds, and extension at 75 °C for 15 seconds. A final extension was performed at 75 °C for 10 minutes. These conditions were optimized to ensure efficient amplification and accurate quantification of the target genes across all samples.

Statistical analysis

Data were analyzed using GraphPad Prism 5 software (GraphPad Software Inc., San Diego, California, US). From the raw data, the control group was normalized to 100% and the percentage of cell renewal of the samples was calculated in relation to the control.

Relative gene expression was calculated using the 2^-∆∆Ct method, which is widely used for quantifying gene expression changes. In this approach, the Ct (cycle threshold) values of the target gene and the reference gene (β-actin) are determined for each sample. The first normalization is performed by subtracting the Ct value of the reference gene from the Ct value of the target gene in each sample (∆Ct). Then, the ∆Ct of the experimental group is compared to the ∆Ct of the control group to obtain ∆∆Ct. The final relative expression value, calculated as 2^-∆∆Ct, indicates whether the gene expression is upregulated (values >1) or downregulated (values <1) compared to the control.

Statistical analysis for comparison between the groups on gene expression was performed using the student's t-test, with significance set at p<0.05. One-way analysis of variance (ANOVA) with the Bonferroni post-hoc test was used for viability comparisons, maintaining a significance level at p<0.05.

## Results

Cell viability

**Figure 2:**
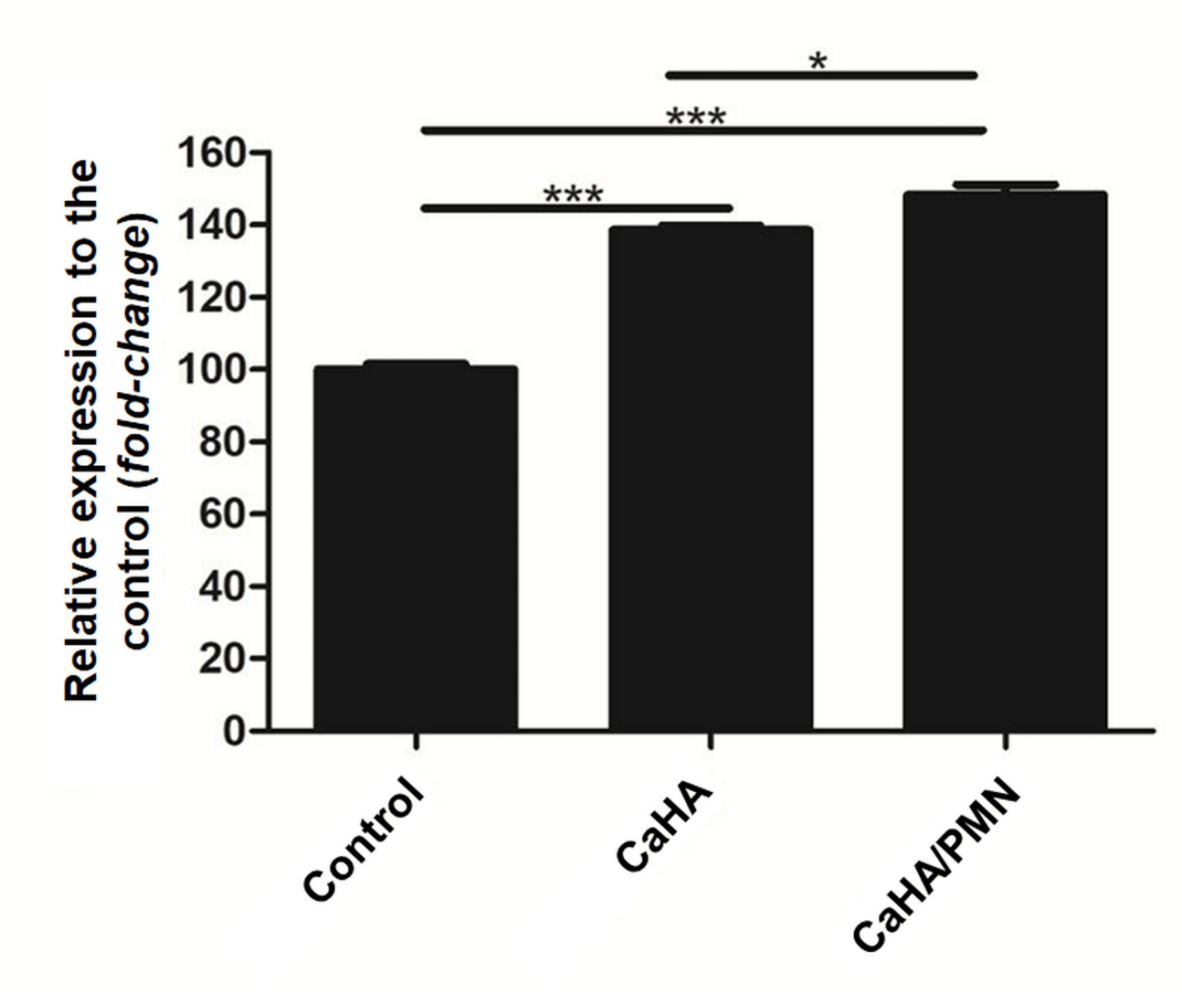
Percentage of cell viability of human fibroblasts after 48 hours of exposure to CaHA and CaHA/PMN samples compared to the control group (*p<0.05; ***p<0.001) CaHA: calcium hydroxylapatite; PMN: poly-micronutrient

Both experimental groups demonstrated a significant increase in cell viability relative to the control (normalized to 100%, ±2.58). The CaHA group exhibited a 38.6% (±2.13) increase while the CaHa/PMN group showed a 48.3% (±4.7) increase. Statistical comparisons revealed significant differences across all groups (p<0.05, p<0.001), with the CaHA/PMN group showing a 6.99% increase compared to CaHA alone (Figure 2).

Gene expression analysis

Significant upregulation of collagen type I and elastin gene expression was observed in treated groups compared to controls. Additionally, treatments induced differential expression of FOXO3, SIRT-1, and SIRT-3, supporting enhanced cellular stress response and extracellular matrix (ECM) remodeling.

Collagen type I gene expression

**Figure 3:**
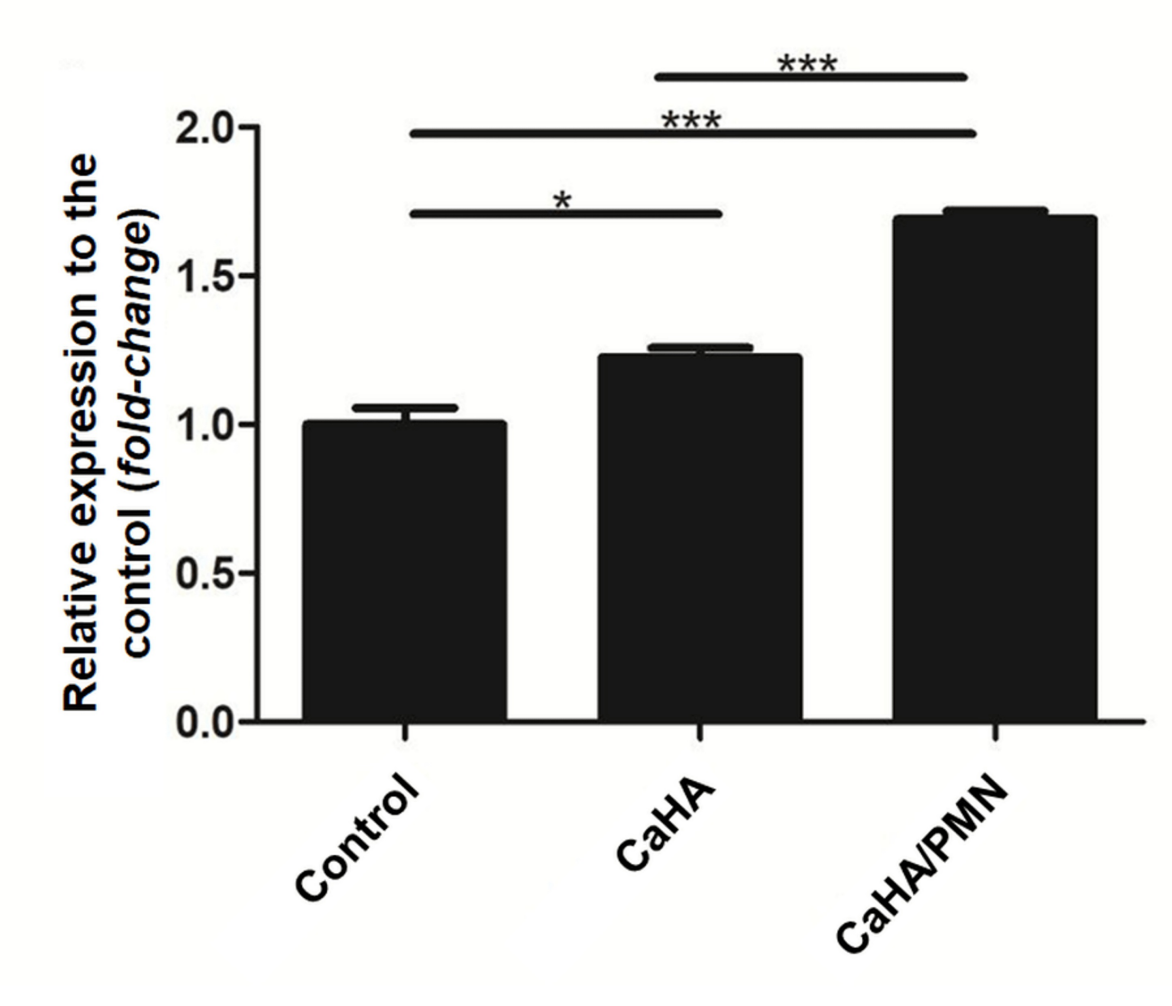
Result of collagen type I gene expression analysis of the evaluated groups Statistical significance is indicated in the horizontal bars (*p<0.05; ***p<0.001).

The CaHA group exhibited a 22.6% (±5.4%) increase in collagen type I gene expression compared to the control group (normalized at 1.0 ± 0.09). While in the CaHA/PMN group, a 69.0% (±4.6%) increase in collagen expression was observed compared to the control. Equating to a 37.8% higher expression than the CaHA group alone. These findings highlight the synergistic effect of poly-micronutrient enrichment on collagen synthesis. Statistical analysis confirmed significant differences across all evaluated groups (p<0.05; p<0.001) (Figure 3).

Elastin gene expression

**Figure 4:**
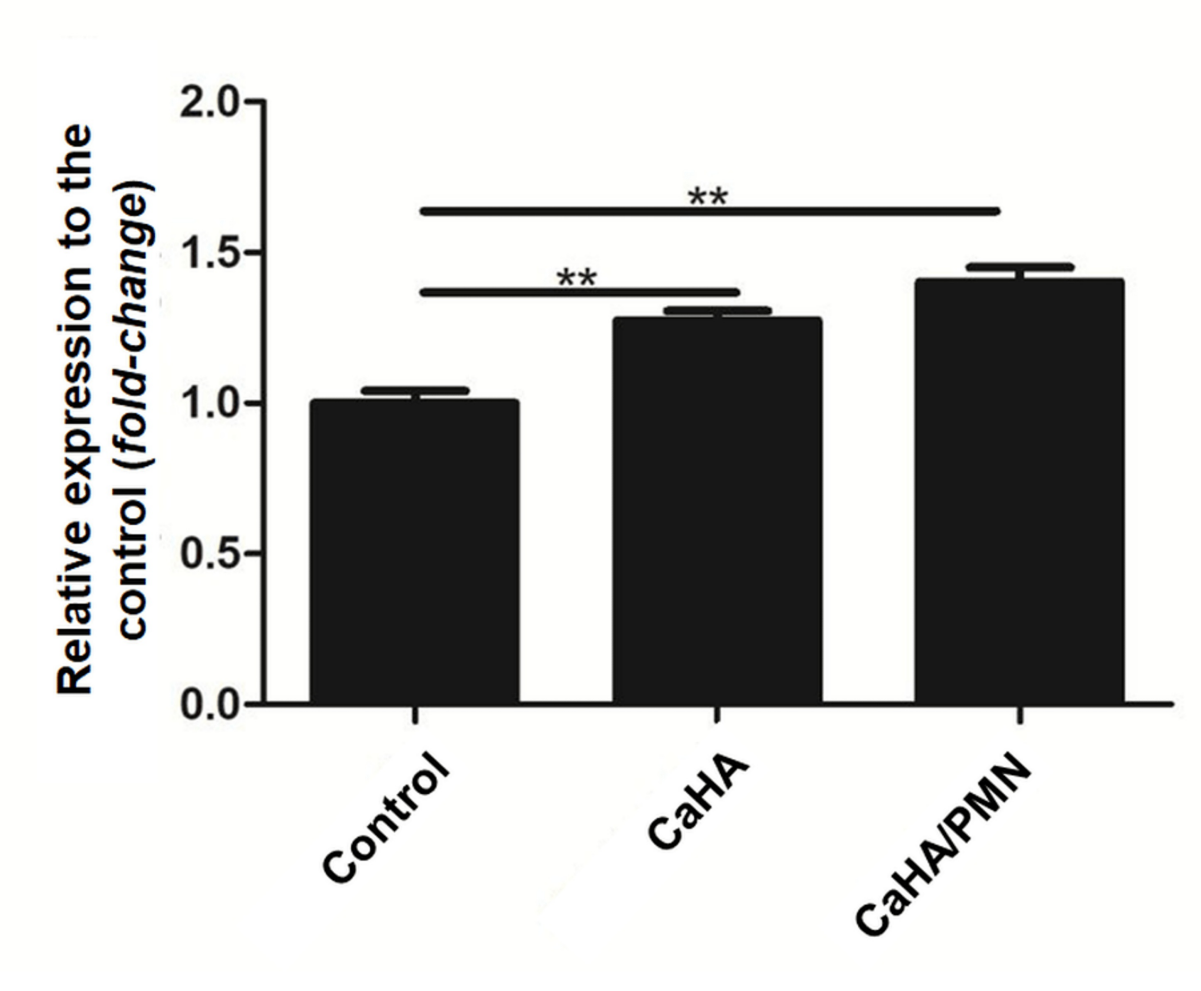
Relative elastin gene expression levels for evaluated groups, with statistically significant differences marked by horizontal bars (**p<0.01)

Cells treated with CaHA exhibited a 27.4% (±5.3%) increase in elastin gene expression compared to the control group. The combination treatment (CaHA/PMN) demonstrated a 40.2% (±8.3%) increase in elastin expression compared to the control group, which was 10.02% higher than the expression observed in the CaHA. Statistical analysis confirmed significant differences across all evaluated groups (p<0.01) (Figure 4).

Longevity-associated genes

SIRT-1

**Figure 5:**
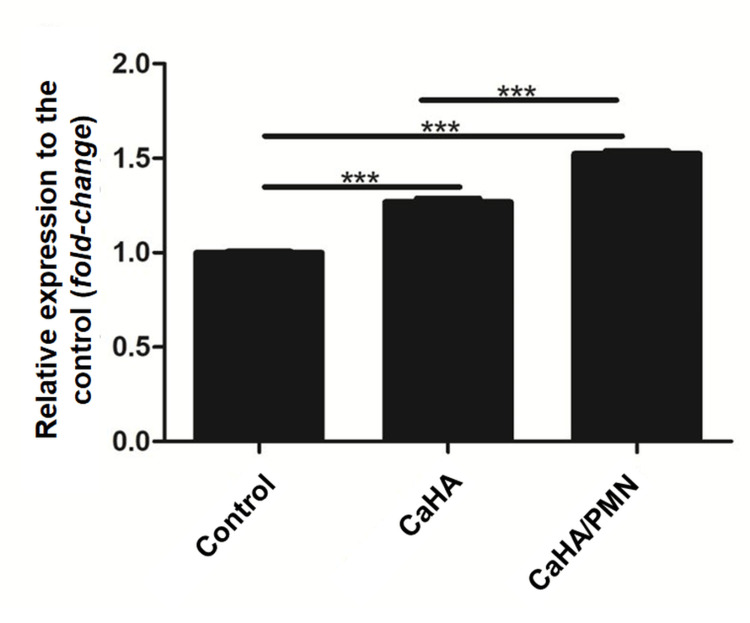
Relative SIRT-1 expression among the evaluated groups, with significant differences denoted by horizontal bars (***p<0.001)

Cells treated with CaHA showed a 26.9% (±3.2) increase in SIRT-1 expression compared to the control while the CaHA/PMN group resulted in a 52.4% (±2.4) increase. When compared, the CaHA/PMN group demonstrated a 20.1% higher SIRT-1 expression than the CaHA group. Significant differences were observed across all groups (p<0.001) (Figure 5).

SIRT-3

**Figure 6:**
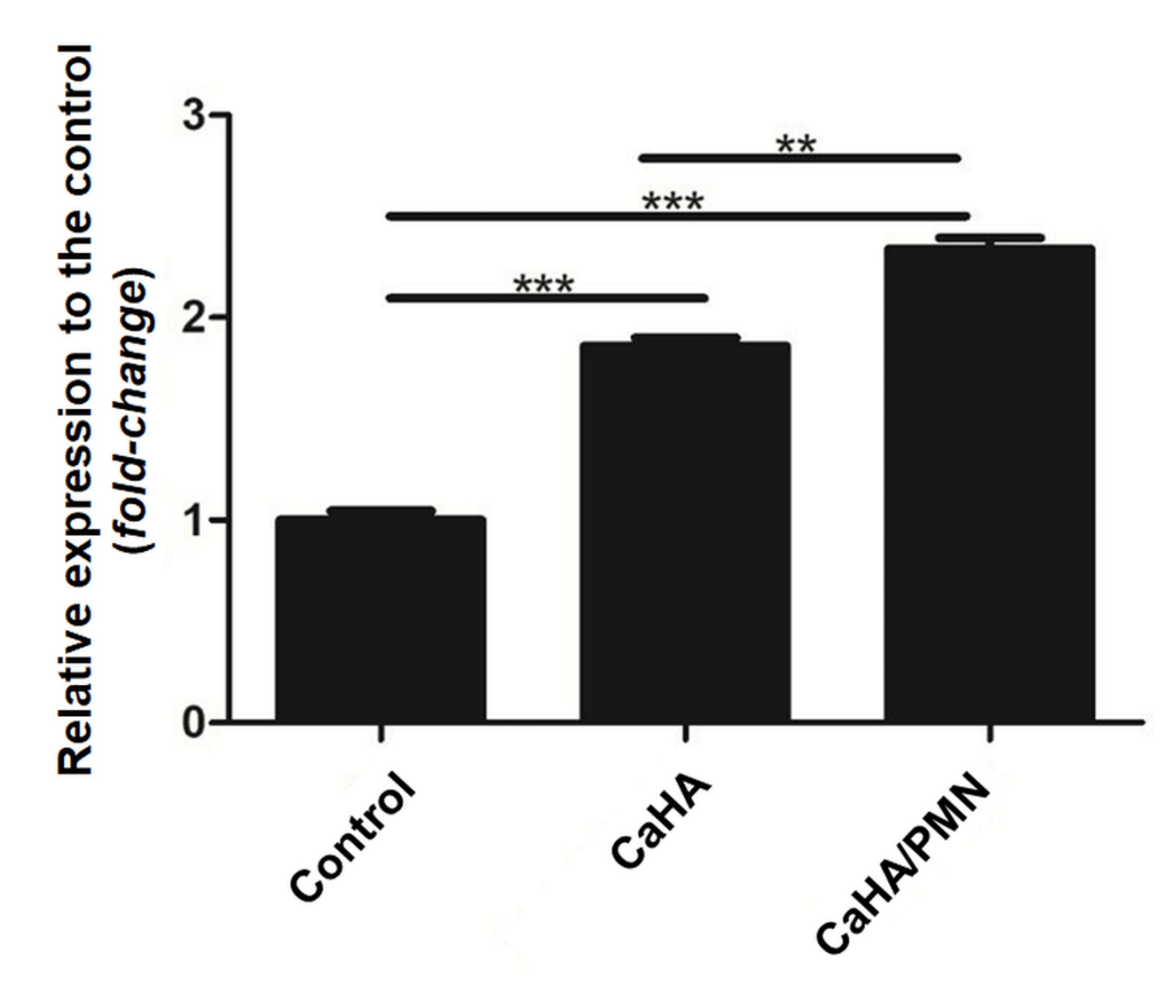
Relative SIRT-3 expression levels in evaluated groups, with statistical significance indicated (**p<0.01; ***p<0.001)

A substantial 86.0% (±7.0) increase in SIRT-3 expression was observed in the CaHA group compared to the control while the CaHA/PMN group showed an impressive 134.0% (±9.4). The CaHA/PMN group exhibited a 25.8% higher expression than the CaHA group alone, with statistically significant differences across all groups (p<0.01; p<0.001) (Figure 6).

FOXO3

**Figure 7:**
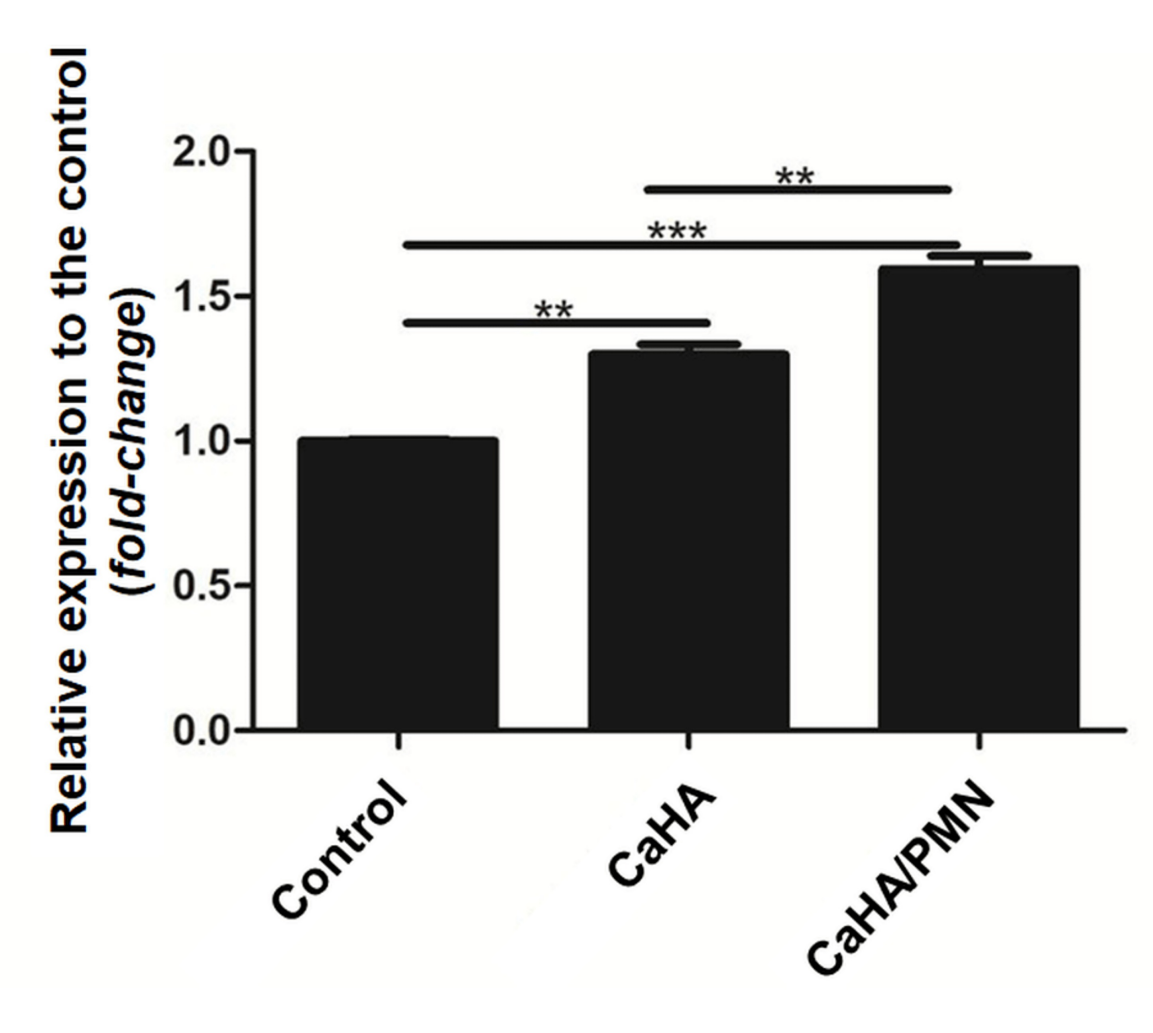
Relative FOXO3 expression levels in evaluated groups, with significant differences represented by horizontal bars (**p<0.01; ***p<0.001)

The CaHA group showed a 29.9% (±6.1) increase in FOXO3 expression compared to the control. The CaHA/PMN group demonstrated a 59.4% (±7.6) increase, surpassing the CaHA group by a 22.7% increase in FOXO3 expression in relation to the CaHA sample. All differences were statistically significant (p<0.01; p<0.001) (Figure 7).

## Discussion

Regenerative aesthetics has revolutionized the way in which skin aging is addressed, shifting the focus from merely concealing signs of aging to actively repairing and rejuvenating skin. This approach leverages the body's inherent regenerative capabilities through advanced biological processes to enhance cosmetic outcomes, offering improvements that are both subtle and sustainable over time [24].

CaHA has been increasingly cited in the literature on regenerative aesthetics as a versatile option [9,25,26]. CaHA is utilized both as a dermal filler and as a biostimulator, being diluted when the goal is biostimulation [6,27]. It promotes the synthesis of key components of the extracellular matrix (ECM), including type I collagen and elastin, which are essential for maintaining the structural integrity and elasticity of the skin [26,28].

Recent studies have highlighted additional regenerative effects of CaHA beyond collagen and elastin production. For example, increased proteoglycan synthesis has been observed following CaHA treatment, a finding that is particularly significant as proteoglycans are critical for tissue hydration and structural support [29,30]. Moreover, CaHA has been associated with angiogenesis and minimal immunological responses, suggesting its broader potential in tissue remodeling and repair [30,31].

CaHA is typically diluted with saline solution and/or lidocaine [6,7]. However, recent studies have also explored dilutions with other substances [32-34]. Among these options, those utilizing solutions enriched with bioactive components stand out, as they may not only enhance the biostimulatory potential of CaHA but also contribute to preparing the extracellular environment for optimal tissue regeneration. By potentially enriching the ECM with essential nutrients, peptides, and growth factors, these formulations create a more favorable microenvironment for fibroblast activation, promoting efficient collagen and elastin synthesis. This optimized ECM remodeling further supports skin regeneration and highlights the potential for innovative approaches in regenerative aesthetics [10].

Building upon these advancements, our study demonstrates not only the already well-established regenerative capacity of CaHA, with a significant increase in collagen and elastin synthesis and enhanced cellular stress response, as evidenced by not only increased SIRT-1, SIRT-3, and FOXO3 expression but also the potential to enhance the results using a poly-micronutrient-enriched solution. These findings further support the potential of PMN to create an optimized microenvironment for skin regeneration and repair.

The combination of CaHA and PMN may improve extracellular matrix protein synthesis, offering potential clinical benefits such as increased skin firmness, elasticity, and enhanced facial rejuvenation and texture. These findings further support the potential of PMN to create an optimized microenvironment for skin regeneration and repair. This concept aligns with clinical evidence reported by Theodorakopoulou et al. [10], who demonstrated that preconditioning the skin with a poly-micronutrient solution enhances the biostimulatory effects of CaHA, leading to improvements in skin firmness, elasticity, and overall facial rejuvenation.

In this context, the roles of molecular markers, such as SIRT-1, SIRT-3, and FOXO3, become particularly relevant. Sirtuin 1 (SIRT-1) is critical in maintaining cellular homeostasis, being involved in oxidative stress response, inflammation regulation, and mitochondrial function. Its activity is also associated with mitigating the progression of several age-related diseases [35]. Similarly, the gene FOXO3 has been consistently linked to longevity across diverse human populations through genetic polymorphisms. FOXO3 contributes to longevity by upregulating target genes involved in stress resistance, metabolism, cell cycle arrest, and apoptosis [36]. Furthermore, FOXOs, including FOXO3, can be post-translationally modified and activated in response to oxidative stress, acting as integrators for maintaining cellular resilience and homeostasis [37,38]. Both CaHA and CaHA/PMN demonstrated a significant increase in the expression of FOXO3 and upregulation of SIRT-1 and SIRT-3. However, the complexity of these signaling pathways and their interactions underscores the need for further research to fully understand their biological significance.

The limitations of the study include its use of an in vitro model of primary human dermal fibroblasts, which, while providing controlled experimental conditions, does not fully represent the complexity of in vivo systems or clinical scenarios. The findings have not yet been validated in clinical trials, and further research is necessary to confirm their applicability in aesthetic and regenerative treatments. Additionally, the 48 to 72-hour incubation period may not account for long-term treatment effects. Since extracellular matrix remodeling is a dynamic process that unfolds over extended periods, future studies should incorporate longer observation times to better assess the biostimulatory response. Furthermore, the analysis focused on a limited set of markers, and future studies should explore additional molecular pathways to gain a more comprehensive understanding of the treatments' mechanisms. Another limitation is that this study relies solely on gene expression analysis (RT-qPCR) without confirming protein synthesis. Since mRNA levels do not always directly correlate with protein abundance, future research should incorporate complementary techniques, such as Western blotting or enzyme-linked immunosorbent assay (ELISA) to provide more robust data.

However, a key strength of this study is its controlled in vitro design, which allows for precise assessment of the biostimulatory effects of CaHA with different diluents. By evaluating fibroblast viability and gene expression of key extracellular matrix components and longevity-associated markers, this research provides valuable preliminary evidence supporting the potential benefits of poly-micronutrient solutions in regenerative aesthetics.

## Conclusions

The results of this in vitro study highlight the biostimulatory effects of CaHA and suggest that its dilution in a poly-micronutrient solution may enhance collagen and elastin synthesis. While these findings indicate potential benefits for aesthetic treatments, further clinical research is warranted to validate these results and optimize protocols for patient care.
